# Editorial: Exploring physical activity and sedentary behaviour in physical disability

**DOI:** 10.3389/fresc.2022.1006039

**Published:** 2022-08-17

**Authors:** Jennifer Ryan, Claire Kerr, Cherry Kilbride, Meriel Norris

**Affiliations:** ^1^ Division of Population Health Sciences, Royal College of Surgeons in Ireland, Dublin, County Dublin, Ireland; ^2^ School of Nursing and Midwifery, Queen's University Belfast, Belfast, United Kingdom; ^3^ College of Health, Brunel University London, Uxbridge, London, United Kingdom

**Editorial on the Research Topic** Exploring physical activity and sedentary behaviour in physical disability By Ryan J, Kerr C, Kilbride C, Norris M. (2022) Front. Rehabilit. Sci. 3:1006039. doi: 10.3389/fresc.2022.1006039

Increasing physical activity and reducing sedentary behaviour reduces the risk of premature mortality, cardiovascular disease, cancer, depression, and type 2 diabetes. For children and adults with physical disabilities (1–5), benefits on function and community participation may also accrue (6, 7). On average, children and adults with physical disabilities are less active than people without disabilities and have higher levels of sedentary behaviour (5, 8). Guidelines recommend that adults with disabilities participate in at least 150 min of moderate activity per week and children participate in at least 60 min of moderate activity daily (6, 7). Supporting children and adults with physical disabilities to increase physical activity and reduce sedentary behaviour may enhance community participation, improve health, and reduce health and social inequalities (6, 7). However, influences on physical activity and sedentary behaviour are multi-faceted and interdependent (9, 10). This research topic aimed to explore the interactions between individual, social and structural factors that influence physical activity participation and sedentary behaviour among children and adults with physical disabilities. In doing so, we aimed to further knowledge and understanding of associations between physical activity, sedentary behaviour, community participation, and physical, mental, and social wellbeing among people with physical disabilities (including the impact of societal and physical barriers). We also aimed to showcase innovative policy and practice approaches to enhancing physical activity and reducing sedentary behaviours across the lifespan.

The nine papers included in the research topic were authored by teams from the United Kingdom, United States of America, Australia and the Netherlands. They employed a variety of research designs, and considered various facets of physical activity in adults with mobility disability Morgan et al. (2022), stroke Church et al. (2021), multiple sclerosis Stennett et al. (2021); Lavelle et al. (2022); Fortune et al. (2021) and rare neurological conditions Ramdharry et al. (2021); and in children and young people with physical disabilities Bolster et al. (2021); Sharma et al. (2021); Sansare et al. (2021). Collectively, this research topic provides a snapshot of the breadth and diversity of research in the area and highlights some of the key considerations when developing, implementing and evaluating interventions to increase physical activity for people with disabilities of all ages.

The International Classification of Functioning, Disability and Health (ICF) (11) was employed explicitly or implicitly in many of the papers in this research topic as a framework for describing the experiences and impact of living with physical disability, operationalising domains that interventions might target, and considering outcomes of physical activity interventions. Three papers highlighted the complexity, interdependency and potentially fluctuating nature of the physical, psychological, participatory and contextual challenges faced by this population when considering, or participating in, physical activity Stennett et al. (2021); Ramdharry et al. (2021); Bolster et al. (2021). Detailed consideration of contextual factors when developing physical activity interventions, potentially before function and disability factors, was highlighted in a number of papers Morgan et al. (2022); Stennett et al. (2021); Ramdharry et al. (2021); Bolster et al. (2021); Sharma et al. (2021). Interestingly, Ramdharry et al noted a mismatch between outcome tools reported in the literature, which focused primarily on activity, compared to the participation-focused outcomes of importance articulated by people with rare neurological conditions Ramdharry et al. (2021). Church et al. noted a similar trend in their paper, demonstrating that although body structure and function outcomes were measured in all 15 empirical studies in their rapid review of high intensity training in people with stroke, participation outcomes were only measured in four Church et al. (2021). Taken together, the papers in this research topic use the language of the ICF to articulate the many influences on physical activity. They advocate for theory-driven physical activity interventions that incorporate behaviour change components, take due cognisance of the individual's health status, their environment and their individual goals, and evaluate outcomes of importance to the individual.

Measurement was a strong theme in the papers included in this research topic. As detailed above, an ICF approach was often employed with a strong focus on participation outcomes. However, measurement validity was also addressed. Lavelle et al. demonstrated poor criterion validity of commercially available devices to monitor step-count and activity time in people with multiple sclerosis Lavelle et al. (2022). In addition to the issues this may cause when evaluating effectiveness of physical activity interventions, it also resulted in frustration and distrust amongst wearers, which could potentially negatively impact motivation to be physically active. It appears that there is still a need for development of psychometrically robust, user-friendly methods of objective measurement of physical activity in people with disabilities.

Sustaining participation in physical activity can be challenging and may be strongly influenced by both personal and environmental factors. Sharma et al. used technology to overcome environmental barriers, demonstrating the feasibility and acceptability of an online physical activity intervention for people aged 12–21 years with a physical disability Sharma et al. (2021). In contrast, Morgan et al. reported outcomes from a long-running community-based exercise programme delivered in an accessible community facility Morgan et al. (2022). Bolster et al. also strongly advocated for consideration of the environment in which physical activity interventions are delivered but acknowledged that provision of physical activity “therapy” in the everyday environment is logistically difficult and thus costly within current service delivery models Bolster et al. (2021). This suggests that innovation is required at policy and health systems levels to deliver impactful interventions in a cost-effective manner.

The value of increasing physical activity and reducing sedentary behaviour for everyone is undisputed – we know “why” it is important. For people with disabilities, the “who”, “what”, “where”, “when” and “how” to optimise physical activity participation are still up for discussion. This research topic demonstrates this complexity but also the innovation and variety in design, methods, implementation and evaluation of physical activity interventions for people with disabilities. It also provides a stimulus for further research in this important area.
